# Speech and Swallowing Data in Individual Patients Who Underwent Glossectomy after Prosthetic Rehabilitation

**DOI:** 10.1155/2016/6548014

**Published:** 2016-11-15

**Authors:** Viviane de Carvalho, Luiz Ubirajara Sennes

**Affiliations:** ^1^Rehabilitation Department, Cancer Institute Arnaldo Vieira de Carvalho, São Paulo, SP, Brazil; ^2^Department of Otorhinolaryngology, University of São Paulo, São Paulo, SP, Brazil

## Abstract

Maintaining oral function in patients undergoing glossectomy boosts interventions such as prosthetic rehabilitation. However, current literature still fails in the presentation of results of prosthetic rehabilitation in relation to speech or swallowing. The objective of this research is to evaluate the effectiveness of prosthetic rehabilitation on voice, speech, and swallowing in patients undergoing glossectomy by performing a systematic literature review and meta-analysis of individual cases. Studies were identified by relevant electronic database and included all dates available. The criteria used were sample with any *n*; resection due to malignant tumors, restricted to tongue and/or floor of mouth; type of prosthetic rehabilitation; and description of the oral functions outcomes with prosthesis. For the meta-analysis of individual data, associations between the variables of interest and the type of prosthesis were evaluated. Thirty-three of 471 articles met the selection criteria. Results on speech and/or voice and swallowing were reported in 27 and 28 articles, respectively. There were improvement of speech intelligibility and swallowing in 96 patients and in 73 patients, respectively, with prosthesis. Based on the available evidences, this article showed that prosthetic rehabilitation was able to improve oral functions and can be a strategy used with surgical reconstruction in selected cases.

## 1. Introduction

Glossectomy is regarded as one of the most devastating treatments for survivors of oral cavity cancer in terms of quality of life [1, 2]. The tongue is an organ with specific, accurate, and particular functions related to speech, voice, swallowing, and chewing. Its excision implies articulatory prejudice in most consonants and vowels [3], unbalanced resonance with predominant hypernasality [4], alterations in vocal quality [5], swallowing impairments that lead to dysphagia in all phases of swallowing [6, 7], and the impossibility or difficulty of performing mastication [7].

While disease control seems to be the main objective of tongue cancer treatment, maintaining oral function in patients undergoing glossectomy also boosts interventions such as prosthetic rehabilitation with a palatal augmentation prosthesis (PAP) or tongue prosthesis (TP) and surgical reconstruction [8, 9]. However, the current literature still fails to present the results of prosthetic rehabilitation in relation to speech and swallowing. One of the possible motives for such findings is probably that the prosthetic rehabilitation of a patient undergoing glossectomy does not occur on a routine basis in most tertiary health centers. This may be due to the sum of various factors, such as ignorance of this type of treatment, the impact of prosthetic rehabilitation on oral function and the quality of life of the patient [10], a reduced number of specialized professionals in this area, and, finally, the trend in recent years toward performing surgical reconstruction on patients undergoing glossectomy for functional rehabilitation [9, 11, 12].

Functional surgical reconstruction of the tongue—especially when a free flap is used by a trained surgical team—is not always possible [13], requires a greater surgical time [14], and increases the cost of treatment, which are limiting factors to this type of rehabilitation, especially in developing countries. Surgical reconstruction also does not exclude prosthetic rehabilitation, as many patients lose some of their teeth or the alveolar ridge after glossectomy [15, 16]. Thus, both PAP and TP could be prepared and coupled to this dental prosthesis and modified at any time without increasing morbidity in these patients.

However, studies on prosthetic rehabilitation are still scarce with limited and often heterogeneous samples, which are measured using nonstandardized measurements with few objective data. A single literature review—which includes 9 works on glossectomized patients who received PAP—concluded that the effectiveness of this type of prosthesis is supported, but the available scientific evidence is still limited [10].

Thus, in order to bring greater clarity to the matter, the objective of this research is to evaluate the effectiveness of prosthetic rehabilitation on maintaining oral functions (voice, speech, and swallowing) in patients undergoing glossectomy by performing a systematic literature review and meta-analysis of individual cases.

## 2. Material and Methods

### 2.1. Searching

Studies were identified by searching MEDLINE, LILACS (Latin American and Caribbean Literature in Health Sciences), SciELO Brazil (Scientific Electronic Library Online) Cochrane, and PubMed databases.

The following keywords were used: glossectomy and prosthesis or protheses; glossectomy and tongue prosthesis or tongue prostheses; glossectomy and palatal augmentation prosthesis; or palatal augmentation prostheses.

### 2.2. Study Selection

The search was limited to human studies and English or Portuguese publications and included all dates available. The last date of search was February 19, 2015.

The following criteria were used for selecting articles: sample with any *n*; resection due to malignant tumors, restricted to tongue and/or floor of mouth, type of prosthetic rehabilitation used (PAP, TP, or other oral prosthetic device); the time and method of speech, voice, and/or swallowing performed; and oral outcomes after prosthetic rehabilitation.

Studies were excluded if relevant results could not be extracted: articles in which the oral function of patients undergoing glossectomy could not be isolated from those presented in combination with data from patients receiving other surgery and review researches and chapters that discussed the prothesis in patients after glossectomy but did not provide empirical data.

Full texts of all articles relevant to oral functions outcomes following tongue cancer surgery and prosthetic rehabilitation were retrieved. For studies with insufficient data in the title and abstract, the full text articles were obtained and searched. A supplementary strategy was used, reference sections from all the identified studies were searched in other relevant citations.

To address the duplication of items recovered in different bases, the documents originally found in each of them were ordered by the title and the first author and those who appeared more than once were excluded.

### 2.3. Assessment of Study Quality

The level of evidence of articles selected was classified using the guideline “The Oxford 2011 level of evidence” provided by Oxford Medical Center Evidence-Based [17].

Titles and abstracts of all identified articles were independently tested by two researchers to determine the relevance of the studies. Disagreements were resolved by a third reviewer or consensus-based discussion.

### 2.4. Data Extraction

From the studies included, a descriptive analysis of data extracted was presented: author, sample size, age range, study design, type of surgery, methodology for evaluation of oral functions, and main results observed.

### 2.5. Methods of Synthesis

The database with information from the studies was stored in the statistical software SPSS for Windows v. 18. Calculation of frequencies and percentages was carried out for all qualitative variables of the study.

For the meta-analysis of individual data, associations between the variables of interest and the type of prosthesis were evaluated by Pearson's chi-squared test or Fisher's exact test depending on the expected frequencies. The significance level for the tests was 5%.

## 3. Results

In total, 470 articles were found in all databases, and 1 article was found after a manual search. In total, 104 articles remained after excluding redundant articles, and 33 articles remained after applying the inclusion criteria for this study.

Most studies included level IV evidence, and only 1 study included level III evidence [18]. Twenty-five studies were case reports: 23 studies on 1 patient [5, 15, 16, 19–38] and 2 articles with 2 patients [39] and 3 patients [40]. Seven studies were case series (between 4 and 36 patients) [4, 41–46], and 1 study was a case-control study with 5 patients in each group [18].

In total, 127 patients were evaluated in these studies. Seventy-nine patients were men, 20 patients were women, and 28 patients were not identified by sex. Patient age ranged from 23 to 78 years (mean age = 58.3 years). Total or subtotal glossectomy was performed on 75 patients, and partial glossectomy was performed on 52 patients. PAP was indicated for 108 patients, TP was indicated for 13 patients, and PAP associated with TP or a gap-filling device in the mandibular area was indicated for 6 patients.

The surgeries associated with glossectomy, surgical flaps, tumor staging, adjuvant/neoadjuvant therapy, speech therapy, and evaluation time are shown in Tables 1 and 2.

Speech and voice results were reported in 27 studies. The main types of evaluation were perceptual [4, 5, 16, 18, 20–22, 24, 25, 28–31, 36, 38, 41, 43–46], acoustic [4, 18, 21, 31, 35], and subjective (nonstandardized assessment of speech) [20, 23, 26, 27, 32, 37, 39]. Only 5 studies used a combination of perceptual and acoustic analyses [4, 5, 18, 22, 31]. Ninety-six patients demonstrated improvement in speech/voice parameters, 7 patients demonstrated worsening, and 12 patients each demonstrated no changes or these changes were not evaluated.

Twenty-eight studies evaluated swallowing outcomes using objective [20, 24, 25, 27, 31, 38, 40, 44, 46], clinical [15, 21, 22, 29, 30, 32, 33, 36, 37, 41, 42, 45], or subjective assessments [5, 16, 19, 20, 23, 33, 34, 39]. Seventy-three patients demonstrated improvement in swallowing with a prosthesis, 1 patient demonstrated worse outcomes [16], 5 patients demonstrated no changes [46], and 48 patients were not evaluated.

Based on the functional differences between the oral tongue and the base of the tongue, the results were presented, where possible, in accordance with the location of tongue resection [9].

### 3.1. Oral Tongue

Five studies evaluated swallowing [34, 39, 40, 44, 45] and/or speech [18, 35, 39, 44, 45] in patients with resection that was restricted to the oral cavity (10–80% resection). Three studies used videofluoroscopy [40, 44, 45]. With a prosthesis, there was an increase in the amount of material swallowed [40] and a decrease in the oral and pharyngeal transit times for thicker consistencies [44, 45], but these findings were less pronounced in patients with 10–20% oral tongue resection because these parameters were normal [44]. Two studies, which subjectively evaluated swallowing, showed that there was greater comfort and ease in swallowing with a prosthesis [34, 39].

In 3 studies [18, 44, 45], there was an improvement in speech intelligibility scores with a prosthesis, which was better in patients with more extensive tongue resections [39]. Only 1 study evaluated changes in the vowels formants and phonemes in a patient with partial glossectomy and a prosthesis, whose values became closer to those measured preoperatively [35].

### 3.2. Base Tongue

Two articles studied swallowing and speech in 6 patients who underwent base tongue resection, whose extensions ranged from 10 to 90% [41, 44]. There was improvement in the oral transit times for different consistencies with the use of prosthesis, and greater reductions were found in patients with resections of 90% and 50% [44]. In another study, there were no reports of worsening or improvement in swallowing with the use of a prosthesis [41]. The prosthesis resulted in the improvement in the fricative and affricative palatolingual sounds and the speech intelligibility rates of 5 patients, which were higher for patients with a larger tongue base resection (90%) [41, 44].

### 3.3. Oral Tongue and Base Tongue

Eighteen studies evaluated swallowing and speech in glossectomized patients with a prosthesis, and the extent of surgery ranged from hemiglossectomy to total glossectomy. Clinical evaluations were performed in 7 studies to assess saliva swallowing and/or swallowing different food consistencies. Two studies noted an improvement in saliva swallowing [21, 30], 5 studies reported an improvement in the ability to swallow food [15, 22, 28, 29, 37], 1 study reported a reduction in the oral transit time for thin pastes [29], in 2 studies swallowing was conducted when the head was straight [30, 37], and in 2 studies laryngeal clinical aspiration was not detected [36] or reduced [28] with the use of a prosthesis.

One study used cinefluoroscopy [20], and 6 studies [24, 25, 28, 31, 38, 45] used videofluoroscopy to evaluate swallowing. The main outcomes were improvements in the ability to handle foods with different consistencies [20, 24], an increase in the swallowing reflex speed [45], a reduction in the pharyngeal transit time [25, 38], a reduction in dry swallowing [25], pooling in the oral and pharyngeal cavities [25, 31, 38], a reduction in laryngeal elevation [31], and the absence or reduction in laryngeal aspiration [25, 27, 28, 38, 44–46]. In 4 studies [5, 16, 19, 33], swallowing was subjectively assessed by the patients, who judged swallowing to be easier with a prosthesis and reported a greater ability to swallow semisolid foods [5, 19, 33]. In 2 studies [16, 46], 5 patients reported no improvement, and 1 patient reported worse swallowing with a prosthesis and used it only for speech.

Speech outcomes showed that there was an improvement in the intelligibility scores for vowels [21, 22], consonants [18, 22, 25, 29, 30], sentences [24], and spontaneous conversation with a prosthesis [4, 24, 29, 30, 44, 45]. Spontaneous conversational intelligibility varied from markedly unintelligible to nearly normal [16, 18, 20, 26, 29, 41, 46], but there was a reduction in the speech rate and an improvement in the intonation and articulation of sounds [5, 31]. Six studies assessed vocal parameters and observed improvements in resonance [5, 20, 31, 41] and voice quality [5, 20, 41], reductions in jitter and shimmer [5, 31], and improvements in formants [4, 5, 22, 26, 31], sibilants [35], and plosives [5, 22, 26, 31].

### 3.4. Free Flap and Prosthetic Rehabilitation

Seven articles were associated with surgical reconstruction with a free flap and prosthetic rehabilitation in 10 patients [15, 16, 35, 38–40, 42]. Three studies reported an involution or inadequacy of the free flap in the floor of mouth [16, 38, 40]. With a prosthesis, there were improvements in the mobility of the remaining tongue [35], the swallowing of thicker foods [15, 39, 40], speech intelligibility [16, 39, 42], and sibilants frequencies and formants values for vowels, which moved closer to preoperative function [35]. Swallowing did not improve with PAP because it interfered with adaptive swallowing in that patient [16].

### 3.5. Meta-Analysis of Individual Data

A meta-analysis of individual data was performed using data obtained from the 33 studies included in this literature review. Comparisons of the PAP and TP groups were performed according to the type of glossectomy, type of tongue resection, speech samples, type of judge (speech therapist, patient, and listener), and type of swallowing evaluation (clinical, objective, and subjective analyses).

There was a significant association between type of surgery and prosthesis, which was positive for PAP and negative for TP (Table 3).

Table 4 indicates that there was no significant association with the area of resection of the tongue and PAP; however, there was a negative association with TP.

There was a significantly positive association between PAP and significantly negative association with TP to spontaneous speech, monosyllables, consonants, and words (Table 5).

The results showed a significant association between PAP or TP and evaluation by speech therapist (*p* < 0.0001; *p* = 0.014), by the patient (*p* < 0.0001; *p* = 0.006), and by listeners (*p* < 0.001, both tests), which were positive for PAP and negative for TP, respectively.

There was a positive association between objective swallowing evaluation and PAP (*p* = 0.002); no association was observed between clinical or subjective assessment and PAP (*p* = 0.582, *p* = 0.095, resp.). TP demonstrated a negative association with objective (*p* = 0.006) and subjective (*p* = 0.024) swallowing analysis and no association was observed on the clinical evaluations.

## 4. Discussion

Prosthetic rehabilitation in patients who undergo glossectomy aims to improve speech, voice, chewing, and swallowing. In this systematic review of 33 studies, these studies, for the most part, showed improvement in these oral functions.

Speech intelligibility after prosthetic rehabilitation probably improved due to increased articulatory precision [31], reduced speech rate [31], improved vocal resonance and control of loudness [5, 20, 31, 41, 46], decreased variability in acoustic components [5, 31], and the movement of the formant values for vowels [4, 18, 22, 26, 35] and frequency values for sibilants [35] and plosives [26] closer to the preoperative levels.

Most patients demonstrated improvement in swallowing with a prosthesis. The main benefits included the increased ability to swallow thick foods [15, 19, 22, 24, 29, 33, 36, 37, 41], decreased oral and pharyngeal transit times [25, 27, 29, 38, 40, 44, 45], and reduction in the use of compensatory maneuvers during swallowing [19, 26, 27, 34, 37, 41]. Safe swallowing with a prosthesis was also demonstrated since laryngeal aspiration was reduced or absent [25, 27, 28, 36, 38, 46] when using these devices, and food stasis decreased in the oral cavity and oropharynx, which can also indirectly reduce the risk of aspiration and/or laryngeal penetration after swallowing [25, 27, 38, 46].

The objective of this research was to gather all published studies in this field in order to determine the common functional outcomes and perform a meta-analysis. Therefore, a meta-analysis of individual data from 33 studies with similar information on each patient was conducted. There was a significant association between PAP or TP, type of glossectomy, and area of resection. Most patients with partial glossectomy or oral tongue resection received PAP. According to the literature, this area of the tongue is responsible for most of the production of the consonant and vowel sounds, and consequently the indications for these prostheses were likely to increase the contact of the residual tongue with the PAP in order to help the development of compensatory movements in speech and swallowing [8].

However, PAP was especially adapted for use in patients who underwent total or subtotal glossectomy, showing that this prosthesis has been indicated for both surgeries. Extensive tongue resection often leads to modifications in the floor of the mouth, the loss of the inferior alveolar ridge and teeth, and disadvantages in adapting the TP, but such difficulties are not found when using PAP since it is fixed to the superior alveolar ridge or superior dental arch (which are usually preserved) to guarantee the stability of this prosthetic device.

Spontaneous speech, monosyllables, consonants, and words were positively associated with PAP. Spontaneous conversation was evaluated in most studies that used nonstandardized protocols, and spontaneous conversation was probably used more often because it is easier to evaluate and crudely represents how patients will be understood in their daily lives. Monosyllables, consonants, and words were also frequently used in these studies, and it can be inferred that some of the studies in the literature, when quantifying the number and types of articulation errors, sought to make speech judgments less subjective as well as select the type of judge (speech therapist) who would be positively associated with PAP. The choice of expert could have made the judgments more judicious and increased the interrelationships among judges, although the literature has observed that listeners with experience in evaluating voice vary widely in their judgments [47]. Objective swallowing evaluation was positively associated with PAP, which also showed the same attempts to make the data more objective and reproducible in other studies.

There was a negative association between TP and speech samples (spontaneous conversation, monosyllables, and words), as well as between objective and subjective swallowing evaluations and the type of judge (speech therapist, patient, and listeners), probably due to the smaller number of patients with TP.

It can be inferred that the main factors associated with possible interference in functional outcomes related to prosthetic rehabilitation are speech therapy, adjuvant/neoadjuvant therapy, associated surgeries, extent of surgery, and area of tongue resection. These correlations could not be statistically assessed because not all studies presented these data.

Speech and myofunctional therapy were instituted in conjunction with prosthetic treatment in 22 studies. Articulatory and prosodic changes often cannot be eliminated with only the use of a prosthesis, and oral rehabilitation is fundamentally important [18, 23, 28, 34]. Thus, speech and swallowing outcomes in glossectomized patients with a prosthesis may also have been influenced by this rehabilitation.

Adjuvant and/or neoadjuvant therapy, which can impair speech and swallowing due to xerostomia, thick saliva, and trismus, did not prevent improvements in the speech and swallowing functions of the patients who had these treatments [22, 24, 34, 39]. Unfortunately, it is difficult to determine if functional outcomes would be even better without these interventions.

Glossectomy was most associated with mandibulectomy, which can, depending on the location and extension, reduce the mouth opening and lead to mandibular deviation and increase articulatory imprecision and nasal resonance; these are speech and voice patterns that already develop in patients after glossectomy. Only 1 study in this review reported slight improvements in speech intelligibility and second formant (F2) of vowels in patients who underwent glossectomy with mandibulectomy and received PAP [4].

Regarding the extent of surgery, it was observed that patients with greater resections—regardless of the area of tongue resection—who initially demonstrated lower scores in speech intelligibility or swallowing also benefited the most from using a prosthesis. Six of 7 patients who demonstrated worsened speech with a prosthesis also underwent partial glossectomy. Meyer Jr. et al. [27] and Cantor et al. [43] observed that patients with moderate restriction of the tongue demonstrated a decrease in speech intelligibility with a prosthesis, suggesting that these patients were able to perform compensatory articulation and the insertion of the prosthesis interfered with, rather than assisted with, their compensatory mechanism.

Swallowing outcomes showed that only 1 patient who underwent total glossectomy worsened in this function with the use of a prosthesis but improved in terms of speech intelligibility [16]. The movements of the orofacial structures differ during speech and swallowing. Thus, the prosthesis may be molded to favor 1 function over the other. Another fact is that after surgery the patient begins to adopt compensatory mechanisms with the remaining muscles of the oral cavity and oropharynx, which are intensified during oral rehabilitation depending on the time of insertion of the prosthesis. Such mechanisms could have already consolidated in this 1 patient, and the prosthesis, when inserted, could have limited such compensations.

Another important aspect of this study was the correlation between the use of a free flap and prosthetic rehabilitation. In recent years, there has been an increase in use of isolated free flaps to improve oral function in patients undergoing glossectomy. However, in the 7 studies included in this review [15, 16, 35, 38–40, 42], the prosthesis was complementary to the surgical flap and improved functional parameters. A common problem with a free flap is the involution of its volume, which increases the gap between the floor of mouth and the hard palate [38, 40], obliterates the mandibular ridge and thereby favors food stasis [15], reduces the oropharyngeal opening [16], increases the intraoral pressure required for bolus transport, and makes it difficult to reconstruct the mandibular region (especially anteriorly), thereby reducing the support of the lower lip [16]. Thus, it was noted that prosthetic devices helped to reduce the intraoral gaps that were not fully resolved by the free flaps, thereby expanding the compensatory strategies of the remaining maxillofacial structures, helping to achieve the proper positioning of the lips, and improving, in most cases, aspects of communication and eating.

It is important to maintain good communication between the head and neck surgeon and the maxillofacial prosthodontist since oftentimes the success of prosthetic rehabilitation depends on certain surgical considerations [10, 16, 46]. The literature shows that some surgeons are unaware of or do not value prosthetic rehabilitation [10], which occurs more frequently in tertiary centers with a maxillofacial prosthesis department, thus giving preference to surgical reconstruction as a form of exclusive rehabilitation for speech and swallowing. It is necessary to understand that competition between these 2 types of rehabilitation is not necessary, but instead cooperation should be encouraged. Speech pathologists and prosthodontists should work together to fabricate the prosthesis [16, 28]. This would favor the patient's adaptation to the prosthesis, as their functional needs would be met.

The limitations of this study include the absence of randomized studies, most outcomes were based on studies with level IV evidence and heterogeneous samples, few studies with statistical analyses or long-term functional outcomes with the prosthesis were included, and nonstandardized data from included studies, which made it impossible to perform a meta-analysis of voice, speech, and swallowing outcomes in patients with a prosthesis.

It is very difficult to make patient groups more homogeneous among studies due to the particularities related to tumor extent, differences between the extirpation techniques used for various neoplasms, and differences in surgical reconstructions. However, attempts by multicenter randomized trials to compare prosthetic rehabilitation, surgical reconstruction, and both types of interventions using standardized assessments would more objectively clarify the benefits of all these types of rehabilitation.

## 5. Conclusions

Based on the available evidence, this literature review and meta-analysis of the individual cases in the 33 included studies on patients who underwent glossectomy showed that prosthetic rehabilitation can improve voice, speech, and swallowing functions and is a viable strategy for selected cases when used in conjunction with surgical reconstruction.

## Figures and Tables

**Table 1:** Study type and characteristics patients of the included articles.

Author	ST	*N*	Age years	TS	Area of resection	Type of surgery	FLAP	RT/CT	Type of prosthesis
Lehman et al. [19]	CR	1	66	NR	OBT	TG + TL	NR	NR	TP
Moore [20]	CR	1	59	NR	OBT	TG + FOM	ALT	NR	TP
Leonard and Gillis [21]	CR	1	48	NR	OBT	TG	NR	NR	TP
Gillis and Leonard [22]	CR	1	46	NR	OBT	TG	DPF	PORT	TP
Knowles et al. [23]	CR	1	46	NR	NR	PG + FOM + M	NR	NR	PAP
Ballard et al. [24]	CR	1	72	NR	OBT	STG + M + TL	TF	PORT	TP
Davis et al. [25]	CR	1	45	NR	OBT	STG + M + epiglottidectomy	NR	PRERT	PAP
Izdebski et al. [26]	CR	1	65	NR	OBT	TG + M	NR	NR	TP
Meyer Jr. et al. [27]	CR	1	69	NR	NR	PG	NR	NR	PAP
Godoy et al. [28]	CR	1	75	T3	OBT	TG	RFFF	PRERT	PAP
Kaplan [15]	CR	1	72	NR	OBT	TG + TL	SGF	NR	PAP + TP
Shimodaira et al. [29]	CR	1	60	NR	OBT	TG	TFL	PRERT	PAP
Çötert and Aras [30]	CR	1	63	NR	OBT	TG	NR	NR	TP
Martins et al. [31]	CR	1	57	T4	OBT	TG	PMMF	PORT	PAP
Goiato and Fernandes [32]	CR	1	62	T2	OBT	TG	NR	PORT	TP
Pigno and Funk [16]	CR	1	57	T4	OBT	TG	RAM	PRERT	PAP
Penn et al. [33]	CR	1	66	T4	OBT	TG + TL + M	IC	PRERT	TP
Dhamankar et al. [34]	CR	1	51	NR	OT	PG	NR	PORT	PAP
Laaksonen et al. [35]	CR	1	64	NR	OT	PG	RFFF	CT + PORT	PAP
Bachher and Dholam [5]	CR	1	59	T4	OBT	TG	PMMF	PORT	TP
Bhirangi et al. [36]	CR	1	NR	T4	OBT	TG	PMMF	PORT	TP
Sabouri et al. [37]	CR	1	46	NR	OBT	TG	NR	PRERT	TP
Okuno et al. [38]	CR	1	64	T2	OBT	STG	ALT	CT + PRERT	PAP + TP
Abdulhadi [39]	CR	2	51,5	NR	OT	PG	RFFF	CT + PRERT	PAP
Koyama et al. [40]	CR	3	62	T4	OT	PG	RAM	NR	PAP + TP
Lauciello et al. [41]	CR	4	59	NR	3 BOT/1 BT	1 TG/3 PG	1: TF; 2: DPF	PRERT	PAP
Leonard and Gillis [18]	CC	5	42,6	NR	OBT	1 TG; 3 PG; 1 STG associated M + FOM	NR	5 PORT	3: PAP; 1 TP; 1 PAP + TP
Okayama et al. [42]	P	6	65	5: T2, 1: T4	NR	5 PG; 1 STG + M	4 RFFF 1 LD 1 SF	NR	PAP
Cantor et al. [43]	R	10	NR	NR	NR	10 = 5 severe restriction + 5 moderate restriction	NR	NR	PAP
Wheeler et al. [44]	R	10	NR	NR	4 OT/6 BT	2 STG; 8 PG (6: 10–20%; 2: 50% resection) + 9 M + 3 FOM	3 TF	NR	PAP
Robbins et al. [45]	R	10	55,4	NR	8 OT/2 OBT	2 TG; 8 PG	NR	NR	PAP
Weber et al. [46]	R	18	NR	T3/T4	NR	18 TG or STG	PMMC	NR	PAP
De Carvalho-Teles et al. [4]	P	36	53,9	4: T2; 6: T3; 26: T4	6 OT 30 BOT	26 TG; 4 STG; 6 PG + 12 M	35: PMMC 1: PMF	35 PORT	PAP

ST: study type; TS = tumor stage; *N* = sample size; RT = radiotherapy; CT = chemotherapy; PORT = postoperative radiotherapy; PRERT = preoperative radiotherapy; CR = case report; P = prospective; R = retrospective; CC = case control; NR = not reported; OT = oral tongue; BOT = base of tongue; OBT = oral and base of tongue; TG = total glossectomy; PG = partial glossectomy; STG = subtotal glossectomy; TL = total laryngectomy; FOM = floor of mouth; M = mandibulectomy; RFFF = radial forearm free flap; ALT = anterolateral thigh flap; DPF = deltopectoral flap; PMMC = pectoralis major myocutaneous flap; RAM = rectus abdominus microvascular free flap; TF = tongue flap; LD = latissimus dorsi; RFFF = radial forearm free flap; IC = iliac crest; FSG = free skin graft; TFL = tensor fascia latae; SF = scapular flap; PF = platysma myocutaneous flap; PAP = palatal augmentation prosthesis; TP = tongue prosthesis.

**Table 2:** Speech, voice, and swallowing outcomes of the included studies.

Author	Sp Sw therapy	Speech/voice tests	Speech/voice outcomes	Sw tests	Sw outcomes	ET
Lehman et al. [19]	NR	NR	NR	Subjective analysis	Improvement to swallow saliva and semisolids	2 weeks
Moore [20]	Yes	Spontaneous speech	Improvement spontaneous speech, resonance, voice quality	Cine-MRI subjective analysis	Sw of a variety of foods	4 m
Leonard and Gillis [21]	Yes	Vowel intelligibility	Improved vowels from 48% to 64%	Clinical evaluation	Sw of saliva improved	4 m
Gillis and Leonard [22]	Yes	Vowels, consonants; acoustic analysis of vowels formants	Improved: vowels 48 to 64%/; consonant: 82 to 90% and F1, F2, F3	Clinical evaluation	Sw semisolids improved	6–8 m
Knowles et al. [23]	Yes	Spontaneous speech	Improved spontaneous speech	Subjective analysis	Improvement global Sw	IE
Ballard et al. [24]	Yes	Word, sentence intelligibility	Word intelligibility: 56% sentence intelligibility: 84%	VF	Improved Sw soft foods	IE
Davis et al. [25]	Yes	CVC plosives intelligibility	Improvement 20%: /t/ and /d/; 33%: /k/ and /g/	VF swallow liquids	Reduction of pharyngeal transit, dry swallows, oral/ pharyngeal residues	1 year
Izdebski et al. [26]	Yes	Conversational intelligibility and vowels and plosives acoustic analysis	Intelligibility markedly improved. Formants achieved partial transitions. Restoration of high-frequency bursts	NR	NR	IE
Meyer Jr. et al. [27]	Yes	Spontaneous speech	Worsened of Speech Intelligibility PAP interfered tongue residual movements	VF liquid and paste	Increase Sw speed, pool reduced, no aspiration	IE
Godoy et al. [28]	Yes	Spontaneous speech	Articulation with substitutions with approximations of target phonemes	VF	Improving bolus propulsion into the pharynx, aspiration decreased to 5%	IE
Kaplan [15]	NR	NR	NR	Clinical evaluation	Sw soft and solid foods increasing the amount of food placed in the mouth	IE
Shimodaira et al. [29]	NR	Syllables, conversational speech	Speech from unintelligible to adequate syllables from 19% correct to 74%	Clinical evaluation of oral transit	Oral transit time 72 to 27 seconds for thin, Sw of thick made possible	IE
Çötert and Aras [30]	NR	Words	Vowel intelligibility from 41 to 57%; consonant from 71 to 84%.	Clinical evaluation	Saliva swallowed with little effort. Head in a vertical position	1 m
Martins et al. [31]	Yes	Acoustic analysis: vowels and automatic speech	Improved articulation, increase in F1, F2, F3 decrease in jitter, shimmer, NHR, nasal resonance speech rate	VF liquid and thin paste	Reduction of pharyngeal residues and reduction laryngeal elevation	IE
Goiato and Fernandes [32]	Yes	Spontaneous speech	Improvement in speech articulation	Clinical evaluation	Improved masticatory efficiency	IE
Pigno and Funk [16]	NR	Spontaneous speech	From intelligible with careful listening to intelligible although noticeably different	Subjective analysis	Sw had worse PAP interfered adaptive swallow	IE, 6 m
Penn et al. [33]	NR	NR	NR	Subjective analysis	Improvement in Sw of solid foods	2 y
Dhamankar et al. [34]	Yes	NR	The patient did need a speech therapist to increase the clarity of speech	Subjective analysis	Comfortable swallowing	IE
Laaksonen et al. [35]	Yes	Acoustic analysis of vowels /i, I, Λ, u/ and sibilants /s, z, *∫*/	Vowels F1, F2 closer to preoperative level; moderate effect on /s, z/	NR	NR	2 y
Bachher and Dholam [5]	Yes	Continuous speech, acoustic analysis: “ee”, “kaap”, “keep”, “kuup”/phonetically balanced passage	Increase in habitual frequency, voice intensity; decrease in jitter and shimmer and voice was more stable and better resonated	Sw questionnaire based on dietary habits of Indians	Sw from liquids to semisolids	6 m 12 y
Bhirangi et al. [36]	NR	Fricative and palatal sounds intelligibility	Fricative and palatal sounds improved audibly	Clinical evaluation	Liquid diet to semisolids without apparent aspiration	6 m
Sabouri et al. [37]	NR	Spontaneous speech	Improvement in speech intelligibility, before it was unintelligible	Clinical evaluation	Liquids to pureed or blended foods with head in an upright position	1 m
Okuno et al. [38]	Yes	Speech intelligibility scores	Speech intelligibility: PAP 50% to 65% PAP + LAP 50% to 73%	VF liquid	Improved oral transit; reduced oral residues; no penetration/aspiration	IE
Abdulhadi [39]	Yes	Spontaneous speech	Improvement in speech intelligibility	Subjective analysis	Easy swallowing	IE
Koyama et al. [40]	NR	NR	NR	VF (2.5, 5, 7.5 ml of gelatin)	All could propel all three volumes of gelatin	IE
Lauciello et al. [41]	Yes	Spontaneous speech	3 PG: speech intelligibility improved and 1 TG: unable function of speech	Clinical evaluation	Nasogastric tube to liquid or thin liquids	1 m
Leonard and Gillis [18]	NI	Speech intelligibility, consonant scores and F2 vowels	Improvement: consonants 9–21%, F2: 8–21%: IF: 8–22%, better in TG patient	NR	NR	6 m
Okayama et al. [42]	Yes	NR	NR	Sw of saliva and US tongue movement	Duration of lingual movement decreased	38 m
Cantor et al. [43]	NI	Words /K, G/ intelligibility	Severe group: improved +15.8–36 Moderate group: worsened −1.6–10.6	NR	NR	2 weeks
Wheeler et al. [44]	Yes	Spontaneous speech	Speech intelligibility improved: 6–18%	VF liquids, thin paste, thick paste	Oral and pharyngeal time reduced for all foods	4–6 weeks
Robbins et al. [45]	Yes	Target sounds/rainbow passage/spontaneous speech	Improvement in articulation: IE 4.5; after 6 m: 3.4	Oral transit clinical analysis: thin, thick	IE: 3.5/6 m: 2.2 aspiration reduced	IE, 3 m, 6 m
Weber et al. [46]	Yes	Speech spontaneous scale	Speech was good or fair: 7/18 (PAP), 10/18: (PAP + laryngeal suspension)	Clinical and VF	13/18 achieved oral alimentation	NR
De Carvalho-Teles et al. [4]	Yes	Speech spontaneous scores and analysis of formants of vowels	Speech spontaneous scores improved: 8.8 to 9.4. increase in F1, F2, F3 values	NR	NR	9.3 m

Sp = speech; Sw = swallowing; ET = evaluation time; m = months; VF = videofluoroscopy; NR = not reported; F1 = First formant, F2 – Second formant; F3 = Third Formant; PAP = palatal augmentation prosthesis; TP = tongue prosthesis; TG = total glossectomy; PG = partial glossectomy.

**Table 3:** Type of glossectomy and palatal augmentation prosthesis (PAP) or tongue prosthesis (TP).

Type of prosthesis	Type of glossectomy	Prosthesis		*p*value^1^
		No	Yes	
		*N*	*N*	
PAP	Partial glossectomy	3	49	0.015
	Total and subtotal Glossectomy	16	59	
TG	Partial glossectomy	52	0	<0.001
	Total and subtotal glossectomy	62	13	

^1^Fisher exact test; *N* = sample size.

**Table 4:** Area of resection of tongue and palatal augmentation prosthesis (PAP) or tongue prosthesis (TP).

Type of Prothesis	Area of resection of tongue	Prosthesis		*p*value^1^
		No	Yes	
		*N*	*N*	
PAP	Oral tongue	3	16	0.352
	Base of tongue	0	7	
	Oral & base of tongue	16	49	
TP	Oral tongue	19	0	0.042
	Base of tongue	7	0	
	Oral & base of tongue	52	13	

^1^Fisher exact test. *N* = sample size.

**Table 5:** Speech samples and palatal augmentation prosthesis (PAP) or tongue prosthesis (TP).

Speech samples		PAP		*p* ^1^	TP		*p* ^1^
		No	Yes		No	Yes	
		*N* = 15	*N* = 101		*N* = 103	*N* = 13	
Spontaneous speech	No	7	13	<0.001	13	7	<0.0002
	Yes	8	88		90	6	
Monosyllables/consonants/words	No	7	75	0.028	76	6	0.039^2^
	Yes	8	26		27	7	
Vowels	No	9	60	0.965	61	8	0.873^2^
	Yes	6	41		42	5	
Sentences	No	13	90	0.78	92	11	0.612
	Yes	2	11		11	2	

^1^Pearson's chi-squared test; ^2^Fisher exact test. *N* = sample size.

## References

[1] Fang Q.-G., Shi S., Zhang X., Li Z.-N., Liu F.-Y., Sun C.-F. (2013). Assessment of the quality of life of patients with oral cancer after pectoralis major myocutaneous flap reconstruction with a focus on speech. *Journal of Oral and Maxillofacial Surgery*.

[2] Vartanian J. G., Carvalho A. L., Yueh B. (2004). Long-term quality-of-life evaluation after head and neck cancer treatment in a developing country. *Archives of Otolaryngology-Head and Neck Surgery*.

[3] Furia C. L. D., Kowalski L. P., Latorre M. R. D. O. (2001). Speech intelligibility after glossectomy and speech rehabilitation. *Archives of Otolaryngology—Head and Neck Surgery*.

[4] De Carvalho-Teles V., Sennes L. U., Gielow I. (2008). Speech evaluation after palatal augmentation in patients undergoing glossectomy. *Archives of Otolaryngology—Head and Neck Surgery*.

[5] Bachher G. K., Dholam K. P. (2010). Long term rehabilitation of a total glossectomy patient. *Journal of Indian Prosthodontist Society*.

[6] Christensen J. M., Hutton J. E., Hasegawa A., Fletcher S. G. (1983). Evaluation of the effects of palatal augmentation on partial glossectomy speech. *The Journal of Prosthetic Dentistry*.

[7] Pauloski B. R., Rademaker A. W., Logemann J. A. (2004). Surgical variables affecting swallowing in patients treated for oral/oropharyngeal cancer. *Head and Neck*.

[8] Aramany M. A., Downs J. A., Beery Q. C., Aslan Y. (1982). Prosthodontic rehabilitation for glossectomy patients. *The Journal of Prosthetic Dentistry*.

[9] Lam L., Samman N. (2013). Speech and swallowing following tongue cancer surgery and free flap reconstruction—a systematic review. *Oral Oncology*.

[10] Marunick M., Tselios N. (2004). The efficacy of palatal augmentation prostheses for speech and swallowing in patients undergoing glossectomy: a review of the literature. *Journal of Prosthetic Dentistry*.

[11] Matsui Y., Shirota T., Yamashita Y., Ohno K. (2009). Analyses of speech intelligibility in patients after glossectomy and reconstruction with fasciocutaneous/myocutaneous flaps. *International Journal of Oral and Maxillofacial Surgery*.

[12] Woo S. H., Jeong H.-S., Kim J. P., Park J. J., Ryu J., Baek C.-H. (2013). Buccinator myomucosal flap for reconstruction of glossectomy defects. *Otolaryngology—Head and Neck Surgery*.

[13] Nangole W. F., Khainga S., Aswani J., Kahoro L., Vilembwa A. (2015). Free flaps in a resource constrained environment: a five-year experience—outcomes and lessons learned. *Plastic Surgery International*.

[14] Smeele L. E., Goldstein D., Tsai V. (2006). Morbidity and cost differences between free flap reconstruction and pedicled flap reconstruction in oral and oropharyngeal cancer: matched control study. *Journal of Otolaryngology*.

[15] Kaplan P. (1993). Immediate rehabilitation after total glossectomy: a clinical report. *The Journal of Prosthetic Dentistry*.

[16] Pigno M. A., Funk J. J. (2003). Prosthetic management of a total glossectomy defect after free flap reconstruction in an edentulous patient: a clinical report. *Journal of Prosthetic Dentistry*.

[17] OCEBM (2011). *The Oxford 2011 Levels of Evidence*.

[18] Leonard R. J., Gillis R. (1990). Differential effects of speech prostheses in glossectomized patients. *The Journal of Prosthetic Dentistry*.

[19] Lehman W. L., Hulicka I. M., Mehringer E. J. (1966). Prosthetic treatment following complete glossectomy. *The Journal of Prosthetic Dentistry*.

[20] Moore D. J. (1972). Glossectomy rehabilitation by mandibular tongue prosthesis. *The Journal of Prosthetic Dentistry*.

[21] Leonard R., Gillis R. (1982). Effects of a prosthetic tongue on vowel intelligibility and food management in a patient with total glossectomy. *Journal of Speech and Hearing Disorders*.

[22] Gillis R. E., Leonard R. J. (1983). Prosthetic treatment for speech and swallowing in patients with total glossectomy. *The Journal of Prosthetic Dentistry*.

[23] Knowles J. C., Chalian V. A., Shanks J. C. (1984). A functional speech impression used to fabricate a maxillary speech prosthesis for a partial glossectomy patient. *The Journal of Prosthetic Dentistry*.

[24] Ballard J. L., Kerner E., Tyson J., Ashford J., Rees R. (1986). Adenocarcinoma of the tongue complicated by a hemimandibulectomy: soft tissue support for a tongue prosthesis in an edentulous glossectomy patient. *The Journal of Prosthetic Dentistry*.

[25] Davis J. W., Lazarus C., Logemann J., Hurst P. S. (1987). Effect of a maxillary glossectomy prosthesis on articulation and swallowing. *The Journal of Prosthetic Dentistry*.

[26] Izdebski K., Ross J. C., Roberts W. L., Boie R. G. D. (1987). An interim prosthesis for the glossectomy patient. *The Journal of Prosthetic Dentistry*.

[27] Meyer J. B., Knudson R. C., Myers K. M. (1990). Light-cured interim palatal augmentation prosthesis. A clinical report. *The Journal of Prosthetic Dentistry*.

[28] Godoy A. J., Perez D. G., Lemon J. C., Martin J. W. (1991). Rehabilitation of a patient with limited oral opening following glossectomy. *The International Journal of Prosthodontics*.

[29] Shimodaira K., Yoshida H., Yusa H., Kanazawa T. (1998). Palatal augmentation prosthesis with alternative palatal vaults for speech and swallowing: a clinical report. *The Journal of Prosthetic Dentistry*.

[30] Çötert H. S., Aras E. (1999). Mastication, deglutition and speech considerations in prosthodontic rehabilitation of a total glossectomy patient. *Journal of Oral Rehabilitation*.

[31] Martins M. V. G., Vale-Prodomo L. P., Carrara-de Angelis E. (2005). Effect of palatal augmentation prosthesis in swallowing and speech articulation in a patient submitted to total glossectomy: case report. *Revista Fonoaudiologia Brasil*.

[32] Goiato M. C., Fernandes A. Ú. R. (2005). Lengua protética articulada para paciente glosectomizado. *Acta Odontológica Venezolana*.

[33] Penn M., Grossmann Y., Shifman A., Taicher S. (2007). Implant-retained feeding aid prosthesis for a patient following total glossectomy and laryngectomy: a clinical report. *Journal of Prosthetic Dentistry*.

[34] Dhamankar D., Mahadevan J., Gupta A. (2008). Rehabilitation of a patient with partial glossectomy. *Journal of Indian Prosthodontist Society*.

[35] Laaksonen J.-P., Loewen I. J., Wolfaardt J., Rieger J., Seikaly H., Harris J. (2009). Speech after tongue reconstruction and use of a palatal augmentation prosthesis: an acoustic case study. *Canadian Journal of Speech-Language Pathology and Audiology*.

[36] Bhirangi P., Somani P., Dholam K. P., Bachher G. (2012). Technical considerations in rehabilitation of an edentulous total glossectomy patient. *International Journal of Dentistry*.

[37] Sabouri A. A., Safari A., Gharechahi J., Esmailzadeh S. (2012). Prosthodontic rehabilitation for total glossectomy with a magnetic detachable mandibular tongue prosthesis: a clinical report. *Journal of Prosthodontics*.

[38] Okuno K., Nohara K., Tanaka N., Sasao Y., Sakai T. (2014). The efficacy of a lingual augmentation prosthesis for swallowing after a glossectomy: a clinical report. *Journal of Prosthetic Dentistry*.

[39] Abdulhadi L. M. (2012). Different techniques for palatal augmentation in partially glossectomized patients. a report of two cases. *Journal of Aquaculture Research & Development*.

[40] Koyama Y., Ota Y., Sakaizumi K. (2014). Swallowing appliance: Intraoral reshaping prosthesis for dysphagia secondary to oral floor cancer: a pilot study. *American Journal of Physical Medicine and Rehabilitation*.

[41] Lauciello F. R., Vergo T., Schaaf N. G., Zimmerman R. (1980). Prosthodontic and speech rehabilitation after partial and complete glossectomy. *The Journal of Prosthetic Dentistry*.

[42] Okayama H., Tamura F., Kikutani T., Kayanaka H., Katagiri H., Nishiwaki K. (2008). Effects of a palatal augmentation prosthesis on lingual function in postoperative patients with oral cancer: coronal section analysis by ultrasonography. *Odontology*.

[43] Cantor R., Curtis T. A., Shipp T., Beumer J., Vogel B. S. (1969). Maxillary speech prostheses for mandibular surgical defects. *The Journal of Prosthetic Dentistry*.

[44] Wheeler R. L., Logemann J. A., Rosen M. S. (1980). Maxillary reshaping prostheses: effectiveness in improving speech and swallowing of postsurgical oral cancer patients. *The Journal of Prosthetic Dentistry*.

[45] Robbins K. T., Bowman J. B., Jacob R. F. (1987). Postglossectomy deglutitory and articulatory rehabilitation with palatal augmentation prostheses. *Archives of Otolaryngology—Head and Neck Surgery*.

[46] Weber R. S., Ohlms L., Bowman J., Jacob R., Goepfert H. (1991). Functional results after total or near total glossectomy with laryngeal preservation. *Archives of Otolaryngology—Head and Neck Surgery*.

[47] Kreiman J., Gerratt B. R., Kempster G. B., Erman A., Berke G. S. (1993). Perceptual evaluation of voice quality: review, tutorial, and a framework for future research. *Journal of Speech and Hearing Research*.

